# Genome-wide selection by mixed model ridge regression and extensions based on geostatistical models

**DOI:** 10.1186/1753-6561-4-S1-S8

**Published:** 2010-03-31

**Authors:** Torben Schulz-Streeck, Hans-Peter Piepho

**Affiliations:** 1 Bioinformatics Unit, Institute for Crop Production and Grassland Research, Universität Hohenheim, Fruwirthstrasse 23, 70599 Stuttgart, Germany

## Abstract

**Background:**

The success of genome-wide selection (GS) approaches will depend crucially on the availability of efficient and easy-to-use computational tools. Therefore, approaches that can be implemented using mixed models hold particular promise and deserve detailed study. A particular class of mixed models suitable for GS is given by geostatistical mixed models, when genetic distance is treated analogously to spatial distance in geostatistics.

**Methods:**

We consider various spatial mixed models for use in GS. The analyses presented for the QTL-MAS 2009 dataset pay particular attention to the modelling of residual errors as well as of polygenetic effects.

**Results:**

It is shown that geostatistical models are viable alternatives to ridge regression, one of the common approaches to GS. Correlations between genome-wide estimated breeding values and true breeding values were between 0.879 and 0.889. In the example considered, we did not find a large effect of the residual error variance modelling, largely because error variances were very small. A variance components model reflecting the pedigree of the crosses did not provide an improved fit.

**Conclusions:**

We conclude that geostatistical models deserve further study as a tool to GS that is easily implemented in a mixed model package.

## Background

Genome-wide selection (GS) is a marker-based method that predicts breeding values on the basis of a large number of molecular markers, which typically cover the entire genome [1]. The idea is to estimate the effects of all genes or chromosomal segments simultaneously and to integrate these estimates in order to predict the total breeding value.

One basic approach for GS is ridge regression (RR) [1]. An interesting alternative to RR is to use spatial models [2] to model genetic correlation among relatives [3].

This study compares RR models and spatial models for estimating genome-wide breeding values for the common dataset provided by the 13^th^ QTL-MAS workshop. The focus is on methods that can be easily implemented using a standard mixed model package with facilities for spatial covariance structures.

## Methods

### Data

The dataset was simulated as part of the 13^th^ QTL-MAS workshop (see [4] for details). Phenotypes of 1000 of 2025 individuals were recorded at five different times (0, 132, 265, 397 and 530), so there is a series of five repeated measurements for each phenotyped individual. Breeding values for the non-phenotyped individuals were to be predicted for time=600, which constitutes an extrapolation.

### Extrapolation for time=600

Careful inspection of the data revealed that a logistic model

would give a reasonable fit to the data, where* y*_*it*_ is the trait value of the *i*-th individual at time* t *(*t* = 0, 132, 256, 397, 530 or 600) and *α_i_*, *β_i_*, *γ_i_* are parameters pertaining to the *i*-th individual. Observed data were modelled as* y*_*it*_* = E*(* y*_*it*_ ) +* ε_it_*, assuming that errors* ε_it_* are independent and have constant variance. We initially fitted this model separately for each individual. Based on this analysis, we then obtained a pooled residual variance estimate from all individuals as =* RSS* /2*n*, where* RSS* is the pooled residual sum of squares and* n* is the number of phenotyped individuals. Each individual contributed two error degrees of freedom. Next, nonlinear regressions were re-run for all phenotyped individuals to predict *y*_*i*600_, fixing the residual variance at the pooled estimate. Along with predictions the standard error was determined. The error variance of predicted values was estimated as the square of the standard error. This variance was subsequently regarded as a known within-individual error variance for mixed model analyses.

### Marker scoring

The marker covariate* z_ik_* for the *i*-th genotype and the* k-*th marker for biallelic markers with alleles* A*_1_ and* A*_2_ was set to 1 for* A*_1_*A*_1_, -1 for* A*_2_*A*_2_ and 0 for* A*_1_*A*_2_ and *A*_2_*A*_1_. Covariates were stored in a matrix*** Z** = *{*z_ik _*}.

### Model

The approach to GS closely follows [2]. Our basic model was

where* µ* is an intercept,* h_i_* is the total genotypic effect of the *i*-th individual and* e_i_* is a residual error. In most cases the total genotypic variance can not be explained by the markers. Thus there is an unexplained part left. We think that this part should be modelled by a polygenic effect. Therefore the total genotypic effect was partitioned into a component explained by the markers (*g_i_*) and a polygenic component (*v_i_*) not captured by the markers. Thus, the total genotypic effect* h_i_* was partitioned as:

[2]. There were two options regarding the model for var(*e_i_*), the variance of* e_i_.* Either we fixed var(*e_i_*) at the squared standard errors of predictions of *y*_*i*600_ obtained from nonlinear regression and assumed heterogeneous residual variance, or we pooled* e_i_* with* v_i_* and thus implicitly included var(*e_i_*) in the variance structure for* v_i_* which is defined as var(***v***) =***I***, where  is the variance component for polygenic effects. It is common to model the polygenic effect by the relationship matrix. But in this case we assumed independent polygenic effects, because no pedigree information on the parents was available. It must be stressed that var(*e_i_*) is strictly a within-individual error variance component that does not comprise between-individual error components. In the present application, these latter error components are effectively confounded with the polygenic effect *v_i_*. By contrast, in plant breeding applications, field replication would allow a separate assessment of between-individual error components. It is conjectured that explicit modelling of such an error component would be of benefit [2], because it is possible that the model captures part of the variance among individuals, which is nongenetic. For this reason it is advisable to generally obtain an independent estimate of error (as in [5]).

We considered different models for the variance of*** g***' = (*g*_1_,* g*_2_,..., *g_G_*), conditionally on the markers ***Z*** = {z*_ik_* }. All conditional models were of the form

for some matrix **Γ** that is a function of*** Z*** and  is a variance component. The models that were used are identical to those used in [2]. Under the mixed model for RR the matrix **Γ** =***ZZ***' was used and the penalty parameter depends on the variance components through *λ*^2^ =  /  , where  is the residual variance [2]. In addition, different spatial models were used. Under these models, the genetic correlation is expressed as

where* d_ii'_* is the Euclidean distance of genotypes* i* and *i*', defined as* d_ii'_=||**z**_i_ – **z**_i'_ ||*, with*** z**'_i_* equal to the* i*-th row of ***Z***, and *f*(*d*) is some monotonically decreasing function of* d*. There are different options for the function *f*(*d*), including those shown in Table 1 [6]. We used all mentioned models and an independent model where  is omitted. It is noteworthy that the quadratic model is equivalent to the RR [2].

**Table 1:** Genotypic covariance models of the form **Γ** = {*f*(*d_ii'_*)}, where* d* is the Euclidean distance computed from marker data and θ is a parameter.

Name	Equation
Linear	
Quadratic	
Power	
Exponential	
Gaussian	
Spherical	

We also considered an extended model , where **Ω** represents the covariance due to simple random effects, i.e., , where ,   and  are the variance components for random effects of father and mother of crosses and of the crosses themselves, respectively, and*** V_f_ , V_m _*** and ***V_c_***  are corresponding symmetric matrices of known constants and  are the variance component for random effects of the individuals. In this case the polygenic variance is defined as , where ***v**^'^= v_1,_v_2_,...,v_G_*. If **Ω** = 0 the model is equal to models with independent polygenic effects. When fitting the extended model we did not fix var(*e_i_*) at the squared standard errors of predictions of* y*_*i*600_, therefore var(*e_i_*) is pooled with .

For each fitted model we obtained BLUPs of *µ* + *h_i_* corresponding to genome-wide estimated breeding values (GEBV). For non-phenotyped individuals   in the case of models with independent polygenic effects and  for the extended model when **Ω** ≠ 0. The Pearson correlation between the GEBV and the fitted values *y_i_*_600_ were calculated. In addition, the Akaike Information Criterion (AIC) was recorded. Small values of AIC indicated a preferable model.

After the 13^th^ QTL-MAS workshop the organizers reported the true breeding values (TBV). The TBV of the non-phenotyped individuals were compared to the GEBV by the Pearson correlation.

### Software

The nonlinear regression was done by the NLMIXED procedure of the SAS System, while all mixed models were fitted by the REML method using the MIXED procedure of SAS.

## Results

### Analysis without fixing the residual variance

First the models were fitted without fixing the residual variance (Table 2). The RR and spatial methods give better fits than the model with independent genotypic effects. The AIC values of the RR and the spatial models are relatively close. The spatial linear, power, exponential and spherical models have a smaller residual variance than the RR/quadratic spatial model and Gaussian spatial model. The latter models show a higher correlation of GEBV with TBV (Table 2). Overall, the RR/quadratic model has the best AIC value, but this model shows a relatively low correlation of GEBV and *y_i_*_600_.

**Table 2:** Model fits of different genetic covariance models and Pearson correlation between GEBV and fitted value and between GEBV and true breeding value (TBV).

		Residual		Correlation	
				
Model for *g_i_*	AIC	variance^§^	*θ*	Fitted value^$^	TBV^#^
**Independent**	6789.1	51.89			
**Ridge Regression (RR)**	6418.5	28.17		0.734	0.889
**Spatial models**					
Linear	6425.8	12.00		0.974	0.880
Quadratic	6418.5	28.17		0.734	0.889
Power	6428.9	12.16	0.99	0.974	0.879
Exponential	6428.5	11.48	216.52	0.977	0.879
Gaussian	6420.5	28.08	124.59	0.737	0.889
Spherical	6427.8	11.96	959.97	0.974	0.880

^§^ The error variance was pooled with that for *v_i_* into a single residual variance.

^$^ The Pearson correlation between GEBV and fitted values (*y*_*i*600_) of the phenotyped individuals.

^#^ The Pearson correlation between GEBV and true breeding values (TBV) of the non- phenotyped individuals.

The fits from the extended models that include **Ω** are shown in Table 3. The AIC values are a little bit higher than in the models without considering the effects of the parents, the independent model being an exception. The ranking of genotypes remains unaltered. Only when  do we find a non-zero variance for mother effects in **Ω** . Throughout, there is a non-zero estimate for the variance of father effects (), while the variance for cross effects () is zero. The correlations of GEBV with TBV are almost the same as those when **Ω** was omitted (Table 3).

**Table 3:** Model fits of different genetic covariance models with random effects for father and mother of crosses and for the crosses themselves and Pearson correlation between GEBV and fitted value and between GEBV and true breeding value (TBV).

		Residual		Father^&^	Mother^&^	Correlation	
						
Model for g_i_	AIC	variance^§^	*θ*			Fitted value^$^	TBV^#^
**Independent**	6605.1	40.77		7.16	6.16	0.481	0.649
**Ridge Regression (RR)**	6420.2	28.18		0.61	0	0.734	0.889
**Spatial models**							
Linear	6427.2	12.17		1.08	0	0.973	0.879
Quadratic	6420.2	28.18		0.61	0	0.734	0.889
Power	6430.3	12.35	0.99	1.20	0	0.973	0.878
Exponential	6429.9	11.62	208.63	1.11	0	0.976	0.878
Gaussian	6422.2	28.07	118.46	0.62	0	0.737	0.889
Spherical	6429.2	12.17	802.96	1.09	0	0.973	0.879

^§^ The error variance was pooled with that for *v_i_* into a single residual variance.

^&^ The estimate of the variance for cross effects  was zero in all models.

^$^ The Pearson correlation between GEBV and fitted values (*y_i_*_600_) of the phenotyped individuals.

^#^ The Pearson correlation between GEBV and true breeding values (TBV) of the non- phenotyped individuals.

### Analysis with fixing the residual variance

The results of the models with a fixed residual variance at the squared standard errors of predictions of *y_i_*_600_ obtained from nonlinear regression are shown in Table 4. The AIC values show that the models with an independent estimate of error var(*e_i_*) have an equal or nearly equal fit compared to the models without fixing the residual variance (Table 4). The correlations of GEBV with TBV are almost the same as those without fixing the residual variance (Table 4). Overall, the RR/quadratic model had the best AIC value as was the case when var(*e_i_*) was not fixed.

**Table 4:** Model fits of different genetic covariance models. Residual variance var(*e*_i_) fixed at value of squared standard error of* y_i600_* and Pearson correlation between GEBV and fitted value and between GEBV and true breeding value (TBV).

		Polygenic		Correlation	
				
Model for* g_i_*	AIC	variance	*θ*	Fitted value^$^	TBV^#^
**Independent**	6789.1	51.80			
**Ridge Regression (RR)**	6418.5	28.09		0.734	0.889
**Spatial models**					
Linear	6425.8	11.88		0.974	0.880
Quadratic	6418.5	28.10		0.734	0.889
Power	6428.8	10.38	0.99	0.981	0.878
Exponential	6428.2	11.17	627.76	0.977	0.879
Gaussian	6420.5	27.98	124.10	0.737	0.889
Spherical	6427.8	11.87	959.00	0.975	0.880

^$^ The Pearson correlation between GEBV and fitted values (*y_i_*_600_) of the phenotyped individuals.

^#^ The Pearson correlation between GEBV and true breeding values (TBV) of the non- phenotyped individuals.

## Discussion

There are only minor differences of the AIC values between RR and spatial models, like in [2]. Thus, some of the spatial models are viable alternatives to RR. Among the spatial models, the Gaussian model gave almost the same fit as RR. This can be explained by a Taylor expansion argument. The correlation function under the Gaussian model is exp(– *d*^2^ /* θ^2^*). When* θ* is large, then the exponent is close to zero and to first order we have , so the Gaussian model approaches the RR/quadratic model in this case, but when* θ* is small we expect different fits. It is noteworthy that the Gaussian model is essentially equivalent to reproducing kernel Hilbert spaces regression as proposed by [7] and also to least squares support vector machine (LS-SVM) regression [8]. It may be conjectured that when inheritance is not merely additive it may be of particular importance to model the genetic covariance by some non-linear spatial model such as an exponential or a Gaussian model.

In this study we have used AIC defined as AIC = -2 log(*likelihood*) + 2 * *number of parameters* as printed by mixed model packages such as the MIXED procedure of SAS. The models with the lowest AIC values showed the highest correlation between the GEBV and TBV. But the correlation between the model ranks produced by AIC and by the correlation between GEBV and TBV is not perfect. For smoothing methods modifications of the AIC have been proposed (e.g. corrected AIC (AIC_C_: [9]) and different other criteria (e.g. generalized cross-validation (GCV: [10]). The main difference to the AIC is that the complexity of the fitted model is calculated as the trace of the so-called smoother matrix tr(***S_λ_***) described in [11], which relates to the effective degrees of freedom of the fit. It is important to realize that GS may be regarded as a smoothing exercise that replaces observed data (adjusted genotype means) by smoothed fitted values. Thus, model selection criteria developed for smoothing can be a useful extension for selecting a preferable model in GS.

The comparison between GEBV and phenotypes is not a good indictor for accuracy of breeding values, when all individuals are involved in the prediction and no independent validation set is left (Tables 2, 3 and 4). Cross-validation is one option to avoid this problem. The leave-one-out cross-validation procedure is equivalent to the cross-validation criterion, which is related to other selection criteria (AIC, AIC_C_ and GCV) [12]. Therefore one idea is to replace the cross-validation procedure by model selection criteria, which would entail a considerable saving of computing time.

In the present study the data were simulated without polygenic effects. Nevertheless, it is prudent to cater for the case that the total genotypic variance can not be fully explained by the markers alone. We think that this unexplained part should be modelled by a polygenic effect.

We modelled the polygenic effect as independent. Alternatively, one can assume that the polygenic effect is correlated due to the pedigree. We modelled the pedigree of the crosses by variance components, however, reflecting the pedigree did not provide an improved fit in the present case.

We also think it is important to separate the polygenic effect from error in order to avoid overfitting [2]. In the present study, however, fixing the residual variance did not have much of an effect because essentially we only had a within-individual error variance estimate. This ignored between-individual error variance, which is therefore expected to be confounded with the variance component for polygenic effects (). In plant breeding trials, where replication is available, one can separate polygenic effects from non-genetic between-individual errors. We expect that such separation will be crucial to the success of GS approaches in plant breeding.

## Conclusions

Our study has shown that geostatistical models are viable alternatives to RR that deserve further study as a tool to GS. With respect to our analyses for the QTL-MAS 2009 dataset, however, we prefer the RR/quadratic model without fixed residual variance for predicting GEBV.

## Competing interests

The authors declare no competing interests.

## Authors' contributions

TSS participated in the design of the study, performed all analyses and drafted the paper. HPP conceived the study, and participated in its design, and helped in the final editing of the paper.
